# Out of pocket payments and access to NCD medication in two regions in Albania

**DOI:** 10.1371/journal.pone.0272221

**Published:** 2022-08-10

**Authors:** Jonila Gabrani, Christian Schindler, Kaspar Wyss

**Affiliations:** 1 Swiss Tropical and Public Health Institute, Basel, Switzerland; 2 Faculty of Medicine, University of Basel, Basel, Switzerland; University of Brescia: Universita degli Studi di Brescia, ITALY

## Abstract

**Objective:**

The financial burden from noncommunicable diseases (NCDs) is a threat worldwide, alleviated only when good social protection schemes are in place. Albeit the Government in Albania has committed to Universal Healthcare Coverage (UHC), Out-of-Pockets (OOPs) persist. Through this study, we aimed to assess the OOPs related to consultations, diagnostic tests, and medicine prescriptions as self-reported by people suffering from NCDs.

**Methods:**

A household survey was conducted in two regions of Albania. The present analysis includes respondents who suffered from chronic health conditions and consulted a health care provider within the last 8 weeks (n = 898). Mixed logistic regression models with random intercepts at the level of communities were employed in order to assess the association of OOPs with age, gender, urban vs. rural residency, health insurance, marital status, barriers experienced, type of chronic condition(s) and region.

**Results:**

Of those who consulted a provider, 95% also received a drug prescription. Among them, 94% were able to obtain all the drugs prescribed. Out-of-pocket payments occurred throughout the NCD treatment process; specifically, for consultation (36%), diagnostic tests (33%), and drugs purchased (88%). Drug expenditures accounted for 62% of all household expenditures. Respondents with health insurance were less likely to pay for consultation and drugs. The elderly (patients above 60 years old) were less likely to pay for consultations and tests. Those who lived in urban areas were less likely to pay for drugs and consultations. Patients encountering any form of barrier when seeking care had increased odds of OOPs for consultations (OR; 2.25 95%-CI; 1.57; 3.23) and tests (OR; 1.71 95%-CI; 1.19; 2.45).

**Conclusion:**

Out-of-pocket payments by NCD patients principally made up through the purchase of prescribed drugs, remain important. Tackling the high costs of drugs will be important to accelerate the UHC agenda. Here, it is important to raise the population’s awareness on patients’ knowledge of their entitlements to health insurance, and on the current health reforms.

## 1.1 Introduction

The burden from the rise of noncommunicable diseases (NCDs) in ageing populations [1, 2]is a world-wide phenomenon in both high-income [3, 4] and low- and middle-income countries (LMICs) [5, 6]. It causes comparatively high out-of-pocket payments (OOPs), especially in countries which have weak financial protection systems [7, 8] and less consolidated primary health care (PHC) services. OOPs are direct (at the point of service) financial contributions, or payments, by patients and their families associated with consumption of medical products (such as medicines) and/or laboratory testing services. They can be formal as well as informal [7–10].

Treatment of NCDs, such as diabetes, cardiovascular diseases (CVD), chronic respiratory diseases (CRD) and cancer often put stress and constraints on household budgets, and can push households into poverty [3]. There is evidence that high OOPs are often related to the purchase of medicines, which can range anywhere from 70% of total health expenditures (in several LMICs) down to less than 10% (in some high income countries).

Moreover, people with one or several NCDs are at times unable to access healthcare due to barriers encountered when seeking healthcare, during treatment, or when trying to get medication [4, 6, 11]_._

Different systematic reviews show that the main access barriers to health care among people with NCDs are: distance of health care facilities [6, 12, 13], lack of adequate public transportation (especially in rural areas) [13], affordability, and financial difficulties [14–16].

In addition to access to public and private healthcare providers for consultation, diagnosis and prescription, the availability and access to affordable medicines is a prerequisite of effective NCD management [17].

Thus, it is highly advocated that implementing Universal Health Coverage (UHC) principles will grant ‘health for all’ and release households from substantive OOP payments for health care [5, 8, 18, 19]. More recently, the financial risk protection for households has been reinforced in the Sustainable Development Goals (SDG) agenda, as a means for reducing poverty [20]. There is evidence that social health insurance improves access to health care services and decreases OOPs [3, 7, 8, 10]. At the same time health insurance schemes define a benefit package eligible for reimbursement so that use of services not falling under this package are to be paid out pocket. In many countries consequently OOPs among insured people remain substantial [8]. Opposite, there is consensus that OOPs should not be too high and (i) allow sick persons to consult health services when needed but (ii) be used to control for excess demand/use of health services_._

### Albanian health system and financial protection policy

Albania, a post- communist Western Balkan country, has joined the majority of European countries in viewing the raise of NCDs as an important public health challenge. Ischemic heart disease was the major cause of mortality in 2019, followed by cerebrovascular disease and lung cancer. Healthcare in Albania is still mainly provided by the public sector, but the number of private health providers has strongly increased over the last three decades [21], and they offer a range of services (mostly in urban areas) [22]. It is divided into three levels: primary, secondary and tertiary healthcare services. Economically active persons contribute to health insurance, while state budget funds (which come from general taxation) cover unemployed and retired persons and those in need, making the scheme a solidarity approach for vulnerable populations [23–26].

The basic service package is the key instrument that defines the range of services to be provided at the PHC level for the entire population, regardless of the insurance status. The basic package encompasses: health care (1) in emergency cases, 2) for children, 3) for adults, 4) for women in reproductive health, 5) for the elderly, 6) mental health care, and 7) promotion and health education [27]. However, expensive procedures and drugs are not included in the basic package.

Contracts with public and private providers concerning the provision of health service packages regulate payments from the health insurance scheme to health service providers pays for service. The health insurance fund signs yearly contracts with primary healthcare services, as well as hospitals and provides indications on reimbursable drugs. Additionally, essential diagnostic tests and paramedical services are free of charge in public facilities for insured people who follow the referral system.

Currently, around 60% of the population is enrolled in the health insurance. In contrast, coverage in the poorest quintile is lower, at 50%. People without valid health insurance and people accessing hospital services without a referral pay the full price for outpatient prescribed medicines, diagnostic tests, paramedical services (such as physiotherapy and speech therapy), inpatient care and pay fixed co-payments for outpatient specialist visits [28].

Over the last decade, the Albanian government has committed to implement UHC (ensuring that people have access to the essential health care they need without suffering financial hardship) and has undertaken a series of steps to adhere to this commitment. Since January 2016, roughly 600,000 uninsured Albanians benefit annually from (i) free family doctor visits, (ii) reimbursements of up to 50% of the drugs’ price (determined by the essential drugs list), if they suffer from NCDs and follow the referral system, and (iii) entitlement to free medical check-ups (applying to nearly one million Albanians aged between 35–70 years old, both insured and uninsured).

The share of the OOPs as a percentage of current health spending has decreased from 60,4% in 2000 to 43,3% in 2015, which afterward slightly increased again to 44,6% in 2018. The latest percentage is now higher than the average in OECD (13.2% in 2019) countries and the EU (15.5% in 2019) [29].

A recent study found that, despite of a decrease in OOPs from 72% in 2009 to 66% in 2015, over 12% of households–around 399,000 people–experienced catastrophic levels of spending on health care, defined as out-of-pocket spending exceeding 40% of a household’s non-subsistence spending, in 2015 [28].

While policy measures -under the UHC umbrella- are implemented in different contexts and systems, differences in the effectiveness of such policy measures are of high regional/international interest [20, 30]. Several questions still remain: who among people suffering from NCDs actually pays out-of-pocket and how much do they pay? In which types of facilities are OOPs more likely to happen? Such questions are relevant for most healthcare systems in Western Balkan countries [31] where many patients and their households are exposed to the consequences of OOPs [32–35] and which therefore aim to move towards UHC and integrating NCD services at the PHC level. However, quality data for informing policy are generally lacking in these countries [36].

The present study aims to fill part of this gap and provides information for understanding the OOP patterns in the population suffering from NCDs. More explicitly, it focuses on the likelihood of making out-of-pocket payments for consultations, drugs, and diagnostic tests among insured and uninsured NCD-patients consulting different providers. In particular, we aim to investigate the access to NCD medications, associated OOPs and financial barriers, as self-reported by people suffering from NCDs.

## 1.2 Methods

### Study design and area

The data for this study were collected by the Household Survey within the “Health for All” (HAP) project in Albania, funded by the Swiss Agency for Development and Cooperation [37]. The household cross-sectional survey was conducted in December 2018 in two regions: (1) Fier which is located South-West of the capital, Tirana, with access to the seaside and (2) Diber a mountainous region, located in the Eastern part of the Country bordering on North Macedonia. The study design and area have been described in recent publications [37, 38].

### Study population and sampling

The study population comprised adults aged 18 years and above who reported suffering from NCDs such as hypertension, heart problems (CVD), diabetes, rheumatism, respiratory, diseases of the nervous system, mental health problems, stroke and cancer. Households were included if at least one person in the household suffered from a chronic condition or disability that had lasted for more than 3 months. Within each household, the household head was asked about characteristics of the household and household members, including the health and insurance status.

The sampling was conducted in a two-stage approach, randomly selecting villages within districts and households within villages, while stratifying between urban and rural areas, to obtain representative data for the two selected regions of Albania. In a next step, we randomly selected, in each such household, one adult with a chronic disease to obtain information on the type of illness, diagnosis and health seeking behaviour, barriers encountered, access to medicine and OOP payments. The sample size calculation is described in Gabrani, et al [38]. In the households included in the sample lived 3,799 persons. 1,116 (29.4%) among them suffered from at least one chronic condition. Out of them, 64% were females and 40% were between 18 to 59 years old. The majority of people lived in rural areas (64%). Overall, 86% (958) of the respondents reported having health insurance and this percentage was higher among urban residents. Further information on the characteristics of households is given in a previous article [38].

### Variables and questionnaires

First, respondents were asked if they consulted a healthcare provider within the last 8 weeks. If they did so, then they were asked if they faced difficulties in seeking NCD services, and if so, what kind of difficulty they encountered.

Respondents were also asked about the actual OOP expenditures for the NCD treatment over the last 8 weeks. The OOPs were divided into 4 categories: a) treatment costs- (formal fees for consultation); b) money spent on means of transport; c) money spent on diagnostic tests and d) money spent for drugs. Moreover, patterns of drug prescription and drug procurement by patients were assessed. The fees for transport were only considered in the descriptive analyses. We investigated and analyzed associations of the reporting of out of pocket payments for consultations, tests and drugs with sociodemographic characteristics. We were particularly interested in differences associated with holding an insurance card, living in rural as compared to urban areas, and with consulting lower tier levels of care such as PHC, health posts and policlinics as compared to higher tier levels such as hospitals.

We considered potential vulnerable groups who may be more likely to make OOPs, i.e., patients living in rural areas, suffering from multiple chronic conditions, having lower income, or being older than 60 years. Respondents were categorized into two groups, those with only one chronic condition vs. those with more than one condition. Chronic conditions asked were hypertension, diabetes, heart problems, stroke, cancer, respiratory diseases, mental disorders, diseases of the nervous system and rheumatic diseases.

### Data collection and statistical analysis

Data collection was carried out between December 7 to 20, 2018, by 16 interviewers who were organized in four teams. Each team was headed by a supervisor who was responsible for the organization of the team and quality assurance of the data collection process. Interviewers and supervisors were trained in a two-and-a-half-day course, including a pre-test. Data collection was done using an electronic data capture tool programed in Open Data Kit (ODK) software and the use of tablets [39].

Data were analyzed using STATA, version 14 (Stata Corp, College Station, TX, USA). In the present analysis, we were mainly interested in the likelihood of out of pocket payments for consultations, drugs and tests. Descriptive analyses were stratified by type of residence (urban vs. rural), health insurance status (insured vs. non-insured), age (> = 60 years vs. 18–59 years) and region (Diber vs. Fier) and also involved median values of OOPs in different categories. Mixed logistic regression models with random intercepts at the level of communities were used to assess the association of experiencing OOP (for consultation, drugs and test respectively) with age, sex, income, health insurance, marital status, urban vs. rural residence, chronic condition(s) and region.

### Ethical considerations

The study protocol was approved by the ethics committee of north-western and central Switzerland (EKNZ- Ethikkommission Nordwest- und Zentralschweiz), No. 30 715. Moreover, the study protocol and questionnaires received ethical clearance from the Ministry of Health and Social Protection (MoHSP) of Albania on the 8th of October 2018, Nr. prot.5800.

All the study participants were above 18 years of age., Oral informed consent was obtained from all respondents at the beginning of the interview. The interviewer provided the head of the household with an informational letter on the study’s objectives and additional information on the confidentiality and anonymity of data. The interviewer also stressed that participation in the survey was voluntary and that the respondents could withdraw their participation. Of the 1,371 eligible households, 82 did not consent to participate, generating a response rate of 94%.

## 1.3 Results

### Socio-demographic characteristics of respondents

In total, 898 respondents (81%) consulted a health care provider within the last 8 weeks and provided information on prescribed medicines. The subsequent analysis on OOPs was only conducted within this group.

More detailed information on the socio-demographic characteristics of respondents is given in **Table 1**.

**Table 1:** Socio-economic characteristics of patients included the present study.

Total population (n = 898)	%
***Gender***	
Male	37
Female	63
***Age***	
18–59 years	39
≥60 years	61
*Education* *	
None	1
pre-school/ kindergarten	0
primary (grade 1–5)	19
secondary grade (grade 6–9)	48
high school	25
Technical/college	1
University	5
*Source of Income* **	
Private business in Albania	7
Salary	13
Pension	35
Social aid	10
Farming/livestock	18
Remittances	15
Other	2
***Civil Status***	
Married	75
Single/divorced/alone	25
***Health insurance***	
**Yes**	88
No	12
**Benefiting soc. Aid**	
Yes	21
No	79

*54 missing-no answers,

**respondents gave multiple answers

#### Medicine prescription patterns and access to NCD medication over the last 8 weeks

Respondents were asked if any medicines were prescribed to them by the doctor during the most recent visits within the last 8 weeks and if they were able to obtain the prescribed medicines

Nighty five percent of the respondents reported having received a drug prescription during their recent visits of a health service (see **Table 2**).

**Table 2:** Access to medicine and associated expenditures (N = 898).

Variable		Count	Percent
Visited facility over the last 8 weeks (and information on medication obtained)	Yes	898	81
Drugs prescribed by the doctor (4/8 weeks)	Yes	857	95
Availability of drugs at the pharmacy (of n 857)			
	*1. All available*	797	93
	*2. Partly available*	53	6
	*3. Not available*	7	1
Obtaining the available drugs (of n 850)			
	*1. Obtained all*	*797*	94
	2. Partly obtained	46	5
	3. Not obtained	**7**	1
Reasons for not obtaining (procuring **all** the drugs n 53, however respondents mentioned more than one option, n = 66)	Pharmacy not visited (e.g. pharmacy too far away)	2	3
	Could not afford, very expensive	34	52
	Medicines do not have good quality	15	23
	Health status has improved	3	4
	Other	12	18
Payment for obtained drugs (n = 843)			
	*Obtained all for free*	109	13
	*I paid for all the medicines prescribed*	324	38
	*Obtained partly for free and I paid for at least one*	410	49

Of these, 94% were able to get all drugs. In order to obtain these drugs, 38% had to pay for all prescribed drugs, while 49% had to pay for at least one of the drugs and 13% received all the drugs ‘for free’ (100% reimbursed).

Among the patients who were not able to procure at least one drug (making up 6%), lack of money was the most important reason given (52%).

#### Out-of-pocket payment for NCD treatment over the last 8 weeks

The total OOP expenditures were assessed along the main expenditure categories, i.e., consultation fees, (laboratory) diagnostic tests and prescribed medicine. Five percent of respondents reported to have given non-monetary gifts, and the value of these gifts was also obtained. However, such gifts are not further considered.

Up to 64% of all patients suffering from NCDs reported not having paid for health care consultation or visit at the respective health facility over the last 8 weeks and 67% did not pay anything for laboratory tests (**see Table 3**). However, close to 88% paid for drugs. Thirty three percent of those who paid for consultations paid also for the drugs and 91% paid for consultation or drugs.

**Table 3:** Out-of-pocket payment for NCD treatment over the last 8 weeks.

Variable OOP	Consultations %	Tests %	Drugs %
All Respondents (n = 898)	36	33	88
Public (n = 777)	32	29	87
Public and private (n = 86)	55	55	88
Private (n = 35)	71	57	97
PHC (n = 504)	30	28	85
Hospital (n = 354)	39	38	90
Urban (n = 332)	33	35	84
Rural (n = 566)	37	32	90
Insured (n = 786)	33	32	86
Uninsured (n = 112)	52	38	96
Benefitting from SE (n = 187)	30	30	88
Not Benefitting from SE (n = 711)	37	34	87
Adults (n = 352)	43	39	91
Elderly (n = 546)	31	29	86
Diber (n = 434)	35	29	87
Fier (n = 464)	36	37	88

Median expenses by respondent over the past 8 weeks were 28.5 Euro (3500 ALL) for anything related to the treatment of chronic conditions, 19.5 Euro (2400 ALL) for drugs and 1.63 Euro (200 ALL) for transportation. Median expenses on tests and consultations were zero.

The mean of individual shares of drug costs was around 62%, and the respective means were 15% for transportation costs and 23% for both treatment and consultation costs.

#### Out-of-pocket payment by insurance status, residency and type of provider

Overall, 86% of the respondents reported having a valid health insurance and this percentage was higher among urban residents as compared to rural ones (89% vs. 69%) and in Diber as compared to Fier (86% vs. 63% respectively). Health insurance coverage among males and females was comparable (males 74%, females 77% respectively).

The median of total individual costs among patients with chronic conditions was 44.3 Euro (5450 ALL) for uninsured patients and 27.6 euros (3400 ALL) for insured ones.

The percentage of respondents who reported paying for health care consultation was higher among those who were uninsured (52%) than among those with a valid insurance card (33%). The respective percentages of patients paying for drugs were 96% and 86%, respectively.

Of those who held a valid card, 31% paid for both consultations and drugs while 89% paid for consultations or drugs.

Regional differences in the distributions of OOP payments were observed, with median costs for the treatment of chronic conditions in the past 8 weeks being higher in rural settings compared to urban ones (rural: 33.4 Euros, 4100 ALL urban: 24.4 Euros 3000 ALL).

In contrast, the frequency of out-of-pocket payments for health care consultations did not vary by region (35% Diber vs. 36% Fier), or by type of residency, (with 37% among those living in rural areas as compared to 33% among those from urban areas). The same pattern was observed with payments for drugs, with 90% among rural residents as compared to 84% among urban ones. Respondents from the Diber region had lower prevalence of OOP payments in all three components as compared to those from the Fier region, the relative difference being highest with payments for tests (37% vs. 29%).

People attending public facilities only were less likely to pay for consultations than those who attended public and private facilities, the respective percentages being 32% and 55%. Among those who attended solely a private provider, the respective percentage was 71%. The prevalence of OOP-payments for tests was 29% among those attending public facilities only and 57% among those attending private facilities. Among those who attended primary care facilities (i.e., PHC facility, a polyclinic or health post), the frequency of OOP payments was consistently lower than among those attending public hospitals **(see Table 3)**.

#### Out-of-pocket payment by type of NCD

**Fig 1:**
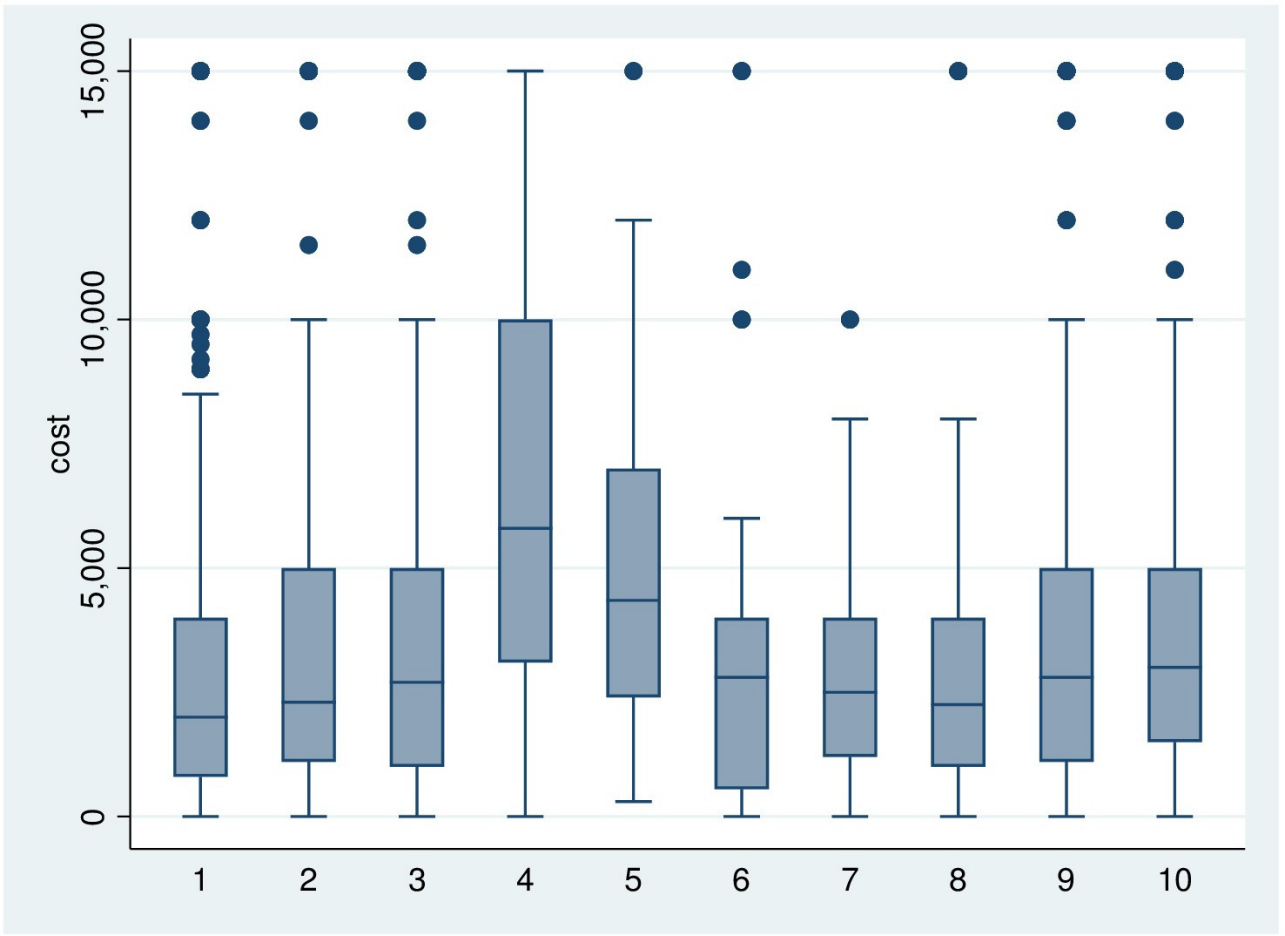
BOX-plot of individual OOP-payments for drugs in the past 8 weeks by type of NCD. Fig 1 shows a box-and-whisker plot of the distribution of the costs of medicines, by type of chronic condition. Respectively: 1. Hypertension, 2. Diabetes, 3. Heart problems, 4. Stroke, 5. Cancer, 6. Respiratory diseases, 7. Mental disorder, 8. Nervous system, 9. Rheumatism, 10. Other Cost in ALL.

**Fig 1** shows a box-and-whisker plot of the distribution of the costs of medicines, by type of chronic condition. The middle fifty percent of the respondents spent between 8 to 33 Euros. For patients with strokes, the median expense was highest (47 Euro), followed by cancer (35 Euro); rheumatic disease and respiratory disease (23 Euro for each).

#### Determinants of out-of-pocket payments for NCD treatment

**Table 4** shows the associations of socioeconomic and socio-demographic determinants (gender, age, socioeconomic beneficiary status, barriers encountered, location of residence, type of chronic condition, health insurance, marital status) with the likelihood of OOP-payments for consultations, tests and drugs.

**Table 4:** Results of mixed logistic regression analyses of specific types of payments.

Variables/Factors N = 898		Any OOP (for drugs, consultations or tests)		Consultation (a)		Drugs (b)		Tests (c)	
		OR	95% CI	OR	95% CI	OR	95% CI	OR	95% CI
Gender	Male								
	Female	1.34	0.79; 2.28	0.99	0.71; 1.37	1.27	0.81; 1.98	1.44*	1.03; 2.01
Age	<60 yr								
	> = 60 yr	0.66	0.35; 1.22	0.65*	0.46; 0.91	0.75	0.45; 1.23	0.66	0.47; 0.93
Marital Status	Married								
	Not Married	0.62	0.36; 1.06	0.76	0.53; 1.10	0.84	0.52; 1.34	0.81	0.56; 1.17
H. Insurance	No								
	yes	0.36	0.11; 1.20	0.51*	0.32; 0.82	0.28*	0.10; 0.81	0.99	0.61; 1.61
Soc-econ.Aid									
	yes	0.92	0.50; 1.69	0.59*	0.40; 0.87	1.01	0.60; 1.71	0.84	0.57; 1.24
Location	Rural								
	Urban	0.75	0.46; 1.21	0.94	0.66; 1.35	0.68^+^	0.45; 1.03	1.16	0.83; 1.62
Barriers seek care									
	yes	1.9	0.93;3.97	2.25 ***	1.57; 3.23	1.61	0.90; 2.89	1.71**	1.19; 2.45
Ch.Conditions									
Hypertension	y vs. n	0.48	0.26; 0.87	0.83	0.59; 1.16	0.73	0.45; 1.18	0.65*	0.46; 0.91
Diabetes	y vs. n	1.56	0.84; 2.90	1.18	0.82; 1.69	1.13	0.69; 1.84	1.68**	1.18; 2.38
Heart problems	y vs. n	1.17	0.71; 1.95	1.58**	1.16; 2.16	1.28	0.83; 1.99	1.33	0.97; 1.82
Stroke	y vs. n	2.08	0.28; 15.63	1.79	0.64;4.97	1.47	0.33; 6.61	4.21**	1.54; 11.48
Cancer	y vs. n	NA**		2.13	0.69; 6.61	NA*		3.29*	1.03; 10.51
Respiratory diseases	y vs. n	0.89	0.37; 2.17	0.84	0.47; 1.51	1.02	0.47; 2.24	1.00	0.56; 1.79
Mental disorder	y vs. n	0.58	0.18; 1.87	2.11**	1.01; 4.43	0.60	0.23; 1.60	0.57	0.25; 1.29
Nervous system	y vs. n	1.68	0.40; 7.04	2.05**	0.97; 4.31	2.61	0.63; 10.87	1.16	0.55; 2.45
Rheumatism	y vs. n	1.29	0.77; 2.16	1.23	0.90; 1.68	1.03	0.67; 1.59	1.28	0.94; 1.76
Fier	vs.Diber	1.29	0.78; 2.13	077	0.54; 1.10	0.93	0.61; 1.43	1.34	0.96; 1.87

*** p < 0.001,

** p < 0.01,

* p < 0.05, + p < 0.1; NA (not available)

NA***cancer variable was removed from model because all 14 cancer patients had to pay for their drugs

Fisher’s exact test of the simple 2 x 2 table between payments for drugs and cancer provides a p-value of 0.24

Insured people were less likely to make any OOP payments (OR: 0.36, 95%-CI; 0.11; 1.20) than those without insurance. Those suffering from hypertension had a lower odds of OOP payments than those without hypertension (OR: 0.48; 95%-CI: 0.26; 0.87). An increased odd of making any OOP was found among those with stroke (OR 2.08, 95%-CI; 0.28; 15.63), conditions related to the nervous system (OR: 1.68; 95%-CI: 0.40; 7.04) and diabetes (OR 1.56; 95%-CI:0.84; 2.90). However, these values are not significant.

Consultations: The odds of consultation payments were lower in the elderly (OR; 0.65; 95%-CI; 0.46; 0.91) compared to the younger adults, in the insured people (OR: 0.51; 95%-CI; 0.32; 0.82) compared to those without insurance and among those profiting from a socioeconomic aid scheme (OR; 0.59; 95%-CI; 0.40;0.87).

On the other hand, the odds of consultation payments were higher among patients with heart problems (OR 1.58; 95%-CI; 1.16; 2.16), mental disorders and disorders of the nervous system.

Insured respondents were less likely to pay out of pocket for drugs (OR: 0.28; 95%-CI: 0.10; 0.81) and the same was observed among those living in urban as compared to rural areas. On the other hand, the respective odds were increased among patients with stroke (OR: 1.47; 95%-CI; 0.33; 6.61) and those with a chronic condition related to the nervous system (OR; 2.61 95%-CI; 0.63;10.87). However, the respective confidence intervals were wide.

Women were more likely to make OOP payments for tests than men (OR;1.44; 95%-CI; 1.03; 2.01). Moreover, suffering from diabetes and stroke, respectively, was associated with a higher odd of making OOP for tests. Elderly people and those suffering from hypertension were less likely to make OOP on tests than their counterparts, with odds ratios of 0.65 (95%-CI; 0.46; 0.91) and 0.66 (95%-CI; 0.47; 0.93), respectively.

When adding barriers as binary predictors (yes/no), we found that patients with barriers had an increased odds of OOP payments for consultations (OR; 2.25 95%-CI; 1.57; 3.23) and tests (OR; 1.71 95%-CI; 1.19; 2.45) compared to patients without barriers. Patients attending a PHC facility were less likely to make OOP payments for consultations and tests compared to those attending a hospital, while there were no significant differences for drugs in the respective comparisons.

## 1.4 Discussion

The main goal of this study was to obtain the self-reported information by respondents suffering from NCDs on patterns of medicine prescriptions, availability and OOPs that followed. A high proportion of respondents reported OOPs for consultations (33%), diagnostic tests (33%) and drugs (89%).

In line with these previous findings, the study results showed a high percentage of drug prescriptions among those who consulted a health service (95%). While availability and accessibility to medicine was plausible, only 11% of respondents obtained their medication ‘for free’. This is consistent with findings from other LMICs, where expenditures for drugs were the predominant component of household health related expenditures [40]. This finding also aligns to recent research in Albania, showing that catastrophic health spending is largely driven by out-of-pocket payments for outpatient medicines [28].

Indeed, the list of reimbursable drugs has been expanded from 1996–2018. Initially, the number of drugs included on the ‘essential’ list was quite low (278 medicines), but constantly grew over the years; 409 in 2008, 477 in 2014, and 540 in 2018. The percentage of co-payments (coverage levels) ranges from 0% to 50% of the reference price. While the reimbursed proportion has substantially increased, the high rate of respondents paying OOPs for medicine provides evidence of a persisting heavy burden. Although some people are exempt (pensioners, people with disabilities, those invalided through war, people with some conditions), there is no explicit exemption from co-payments for people with common chronic conditions or for people with low incomes. There is no overall annual cap (ceiling) on out-of-pocket payments arising from medicines or for other health services. This is especially worrying as co-payments for drugs or tests may accumulate over time [28].

Regarding the phenomenon of high OOPs for drugs, this study suggests the following four explanations: (i) the high percentage of co-payments for outpatient prescribed medicines for people who are covered, [28] leading to high OOPs; (ii) the prescription patterns of the doctors (over- prescribing and, as a consequence, over-consumption of drugs); (iii) prescription of more expensive drugs (that are not on the list of eligible drugs; although the Fund obliges physicians to prescribe the cheapest generic alternatives available); (iv) the populations’ lack of awareness regarding their rights, entitlements and current health reforms. In summary, patients’ spending for drugs might be driven by the doctors’ prescription patterns, coupled with the low awareness of entitlements [28]. Data collection relied on interviews with household members who might not be fully aware of drugs eligible for reimbursement by the Compulsory Healthcare Insurance Fund. Consequently, and albeit highly desirable, investigation on the share of OOPs made up by drugs part of the list for reimbursement -or not- wasn’t achievable by this survey. Consequently, further research into patterns of purchasing drugs would be of interest.

There is a need for the government to monitor the progress of the UHC commitment, in order to ensure that the drugs included in the essential medicine list are being correctly prescribed and dispensed. However, it should be stated that certain higher OOP expenditures for diabetes for example are also likely to be related to the nature of the disease requiring regularly laboratory diagnostics, rather than the all the above explanations.

In most European countries, the ‘*product-specific eligibility’*, or so-called “positive reimbursable list”, is the dominant scheme for drug coverage, complemented by specific rules that grant higher reimbursement to vulnerable groups of population (such as the elderly, low-income households, the disabled, etc.). The Baltic countries use a ‘*disease-specific eligibility scheme’*, meaning that the same medicine may require different co-payments, depending on the disease it is used to treat [41].

In addition to percentage-based co-payment rates that are widespread in the WHO European Region, ‘fixed co-payments’ are also common in several countries (e.g. Estonia, France, Poland). Denmark and Sweden operate ‘*consumption-based reimbursement schemes’*, in which patients have to pay out-of-pocket for medicines, up to a specific threshold of expenses, after which they share payments with the public payer. In Switzerland, the share is 10% of the cost of the prescribed drug. In addition, payments from patients can be required if they refuse the lowest-priced medicine. There are only few countries (e.g. Austria, Croatia, Cyprus–public sector, Germany, Ireland, Italy, Malta–public sector, the Netherlands and the United Kingdom) in which the costs of reimbursable medicines in the public sector are fully covered by the social protection scheme (with no percentage of reimbursement/co-payment applied), but other co-payments may apply (these usually take the form of a prescription fee) [41].

Albania should investigate and analyse which model (or combination of models) is considered to be the *best-practice model* and can ensure access to affordable medicines for the population.

Respondents with health insurance were less likely to encounter OOPs over the last 8 weeks. Nevertheless, 33% of respondents with valid cards reported OOPs for consultations, as compared to 52% of those who were not insured. To some extent, these findings are in line with several other results and policy notes, arguing that health insurance is a sustainable and effective approach for controlling for OOPs [5, 8, 18–20].

Out-of-pocket payments are occurring at all levels of health facilities, from health posts to hospitals. However, in lower -level facilities, such as PHC centers and health posts, respondents were less likely to report OOPs (30% at PHC compared to 51% in polyclinics, and 41% in hospitals). This is confirmed by previous studies conducted in Albania, on different recall periods [28].

Patients in urban areas had to pay less often for drugs and consultations. This might be related to (i) a better organization of the PHC level in urban areas and (ii) a higher awareness and literacy regarding patients’ rights to obtain free generic drugs (from the essential reimbursed list); Moreover, urban residents can more easily access pharmacies [42].

The regression models showed that the elderly were less likely to make any OOPs. This could imply that the elderly are supported at the PHC level, are following the PHC referral system more correctly, and are also following the general rules of family doctors, related to referrals and drug prescriptions. This is consistent with a previous study where the elderly were more likely to initiate and follow up treatment of their NCDs at the PHC level, as compared to adults. Moreover, pensioners are exempt from co-payment for the lowest-priced generic versions of covered medicines. Additional measures to provide full coverage of outpatient medicines for some chronic conditions (based on the lowest-priced option) were introduced in 2017 [28].

Many patients, especially women, paid for tests, although most diagnostic tests and paramedical services are free of charge in public facilities and are covered by health insurance for people who follow the referral system [28]. These findings correlate with our previous observations that people were attracted to the private sector by better medical equipment [21].

The health system’s dependence on private OOPs, including informal, under-the-table payments, has been a concern for the Albanian governments. Consequently, they have tried to tackle this problem by pledging ‘free healthcare’ and by removing the consultation fees at the PHC level and at higher levels for patients following the referral mechanism.

The present study did not address informal payments. Only 5% of patients mentioned informal payments which might be considered a relatively low percentage. On the other hand, there might also be a considerable bias in this number, as many patients might not want to disclose such payments.

However, since the consultations during the past 8 weeks were mostly done at PHC level, the findings cannot be generalized for the other settings (namely, hospitals).

Without changing the population’s knowledge of their rights to health care and their obligations to co-payments, there is a two-fold risk. Firstly, reforms per se are undermined and the “Basic Package of Services” at PHC is wasted. Secondly, if households keep to their old practice of ‘buying services of good quality’, without making use of their entitlements, they are put at risk of catastrophic health expenditures, especially in cases of multiple NCDs whose management requires more financial resources. Thus, defining an explicit package of benefits can help to improve equity in access and increase accountability for the services specified in the package; as patients are aware of the services they are entitled to receive and the respective prices, the scope for payments is reduced [43].

### Limitations of the study

Our information on OOPs relies on self-reports of respondents and thus may reflect subjective perceptions. Unfortunately, we could not identify any means to control for this bias. Additionally, we could not obtain any information on whether drugs prescribed and paid for were on the essential medicine list or not. Consequently, the high out of pocket spending on drugs may include overprescribed, unnecessary drugs, such as vitamins, which are not on the essential list. One area for improvement could consist in adding qualitative interviews or focus group discussions to obtain insights if the OOPs were made for drugs included in the essential list (reimbursed drugs) or for drugs not covered by this list. Thus, we could better understand if patients were overprescribed or prescribed more expensive alternative drugs.

Further, financial information may be perceived as sensitive and there might be either over or under-reporting by any respondent, probably affecting strongly the reporting of informal payments (regardless of the sociodemographic characteristics). The hospital visits might be less frequent than visits at PHCs, so that patients attending mostly hospitals might be under-represented in the present analysis.

The recall period of 8 weeks may have resulted in bias, both for underreporting and overestimation of OOPs. The short recall period might lead to an over-representation of patients with frequent visits and therefore lead to an overestimation of OOPs.

At the same time, underreporting of OOPs, especially for expenditures for drugs or diagnostic tests.

Study findings cannot be generalised to the whole of Albania as the research was conducted in two regions, Fier and Diber, which distinct socio-cultural and economic characteristics and hosting 16% of the overall population of the country. They thus represent OOP patterns as applicable to these regions but cannot be generalised for the whole of Albania, namely for the capital city Tirana where private service providers play an important role.

## 1.5 Conclusions

The OOPs made by NCD-patients largely concern the purchase of prescribed drugs, accounting for more than 60% of expenditures. Consequently, access to adequate treatment and associated costs remain a concern for many households and patients. Reducing access barriers further and addressing issues of high costs of drugs will be important to improve the situation of NCD patients. Continuing efforts to build sustainable a social protection scheme, and to integrate essential NCD services into PHC level, might reduce OOPs and release financial constraints on household budgets.

It is of importance to raise the population’s awareness of patients’ rights, of their entitlements from health insurance and of the current health reforms. Information of patients should be intensified, with a clear focus on the individual patient, and it should emphasize patient involvement in the treatment process, with the aim that patients learn to handle their conditions more adequately.

## Supporting information

S1 Data (CSV)

S2 Data (XLS)
